# Current self-medication practices in the Kingdom of Saudi Arabia: an observational study

**DOI:** 10.11604/pamj.2020.37.51.24098

**Published:** 2020-09-14

**Authors:** Sameer Al-Ghamdi, Tariq Majed Alfauri, Muath Abdullah Alharbi, Mustafa Mohammed Alsaihati, Muhammad Makki Alshaykh, Almuhanad Abdullah Alharbi, Naif Soud Aljaizani, Ibrahim Aidh Allehiby, Matar Abdullah Alzahrani, Abdulsalam Saud Alharbi

**Affiliations:** 1 Department of Family Medicine, College of Medicine, Prince Sattam bin Abdulaziz University, Al-Kharj11942,Al-Kharj, Saudi Arabia,; 2 Dr. Sulaiman Alhabib Medical Group, Sewidi branch, Riyadh, Saudi Arabia,; 3 Faculty of Medicine, Medical University of Lodz, Lodz, Poland,; 4 Faculty of Medicine, Medical University of Gdansk, Gdansk, Poland,; 5 Faculty of Medicine, Prince Sattam Bin Abdulaziz University, Al-Kharj, Saudi Arabia

**Keywords:** Self-medication, nonprescription drugs, over-the-counter drugs, OTC drugs, Saudi Arabia

## Abstract

**Introduction:**

medication without prescription is a growing public health concern or phenomenon worldwide. This cross-sectional study was designed to study the trends of self-medication among Saudi population.

**Methods:**

a prospective, cross-sectional study including 2004 participants was carried out from different family clinics across the Kingdom of Saudi Arabia (KSA). The clinicians used a self-designed questionnaire to collect the data, using stringent inclusion criteria and exclusion criteria. The questionnaire recorded participants' demographics and included several closed-ended and open-ended questions with options to choose from. The results were gathered, inserted into an excel spreadsheet and analyzed using SPSS version 23. The analyzed information was presented as frequencies and percentages.

**Results:**

our research showed that 924 respondents (46.1%) found it difficult to reach a hospital. Another 45.2% considered the inefficiency of health centers in providing necessary care as the main reason for self-medication. Other causes of self-medication included a lack of medical insurance, insurance not covering the costs for the drugs, and symptoms of the disease being mild enough for not going to the hospital. Analgesics were the most commonly self-administered drugs (84.58%), followed by antipyretics (71.26%), cough syrups (46.86%), eye drops (35.98%), antibiotics (35.28%), flu medication (32.83%), heartburn medication (23.15%), medicines for joint pain (15.02%), and so on.

**Conclusion:**

the majority of Saudi Arabians engage in self-medication and rely on advice from friends and family most of the time, instead of consulting a health professional.

## Introduction

Self-medication or medication without prescription refers to the use of drugs for self-diagnosed illness without any consultation of a healthcare provider [1,2]. World Health Organization (WHO) refers self-medication to the process of medicating oneself to treat self-recognized disorders or illnesses [3]. Medication without prescription is a growing public health concern or phenomenon worldwide [4]. Over the globe, billions of health conditions are treated with self-medication every year [4]. However, the prevalence of self-medication varies in different regions of the world ranging from 38.5% to 92% [4]. It represents that a large portion of the world population uses drugs without proper consultation from a doctor or healthcare professional. In developing countries, approximately 80% of drugs are purchased without any prescription [5]. In Saudi Arabia, up to 81.4% of the general population has reported to use drugs without prescription at some point of their life [6]. There are many reasons which give rise to self-medication. Some of the important reasons include limited or no excess to healthcare facilities, timesaving, prior good experience, minor or mild illness, emergency conditions, inexpensiveness, suggestions by friends and sufficient knowledge of drugs [7]. In addition, self-medication may also be promoted by social, cultural and economic factors [8]. Self-medication can be responsible as well as irresponsible. Over-the-counter (OTC) drugs are the most frequently used medicines as self-medication. The US Food and Drug Authority (FDA) endorses OTC medicines as safe and effective for the community without consultation [9]. OTC drugs are well-known and well-accepted practice around the globe [10]. Self-medication with OTC drugs is considered responsible or safe self-medication. On the other hand, self-medication with prescription drugs is irresponsible or unsafe self-medication which may give rise to dreadful outcomes [11].

Self-medication has benefits as well as drawbacks at both individual and community levels. Individual benefits of self-medication include time-saving or rapid access to treatment, convenience, cost-effectiveness and self-reliance in managing minor illnesses. At a community level, self-medication offers economic benefits by lowering the burden on healthcare facilities while saving limited resources and making it possible for people living in remote areas to obtain medicine immediately at lower costs. On the other hand, self-medication may cause damages at both individual and community levels. At individual level, medication without any consultation of medical professional may result in wrong self-diagnosis and treatment, failure to reach appropriate healthcare facility, inadequate dose, wrong route of administration, improper timing of medicine, prolonged treatment, drug interaction, drug toxicity, adverse events, drug dependence, microbial resistance and wasted resources of the country [5]. Similarly, at a community level, medication without prescription may result in the loss of resources and drug-induced medical conditions. Surprisingly, it has been reported that self-medication with prescription drugs is the leading cause of misuse among physicians [12]. Therefore, there is utmost need of proper guidelines about self-medication. Literature on trends of self-medication or medication without prescription is lacking from Saudi Arabia in terms of its frequency, reasons, type of self-medication and associated factors. Therefore, this cross-sectional study was designed to study the trends of self-medication among Saudi population.

## Methods

A prospective, cross-sectional study was carried out on responders from different regions of the Kingdom of Saudi Arabia (KSA) from December 2017 to December 2019. The participants were chosen from patients attending different family clinics across the KSA. The clinicians used a self-designed questionnaire to collect the data. Verbal consent was gained from the participants with an option to decline. The questionnaire was in English, but the questions were translated into Arabic by the clinicians for a better understanding of the participants. The questions were asked in Arabic by the clinicians and the answers were marked by them on the English questionnaire on behalf of the patients. Inclusion criteria included people of all age groups, both genders, residents of KSA who presented to family clinics and provided verbal consent to participate in the study. Patients who denied participation in the study, minors who did not provide legal consent by the guardian, short time visitors to KSA, prisoners, and all cases that needed hospitalization were excluded. The questionnaire recorded participants' demographics and included several closed-ended and open-ended questions with options to choose from. The demographics section recorded participants´ age, gender, educational level, nationality (Saudi vs. non-Saudi), marital status, number of kids, and where they belonged inside the KSA. Other questions included practices regarding self-medication, reasons for self-medication, and the types of medication used. The results were gathered, written on an excel spreadsheet and analyzed using SPSS version 23. The analyzed information was presented as frequencies and percentages. The Chi-square test was used to find the correlation between different factors for using drugs and the types of drugs used against patient demographics as individual variables. P < 0.05 was set as statistical significance for all comparisons.

## Results

**Figure 1:**
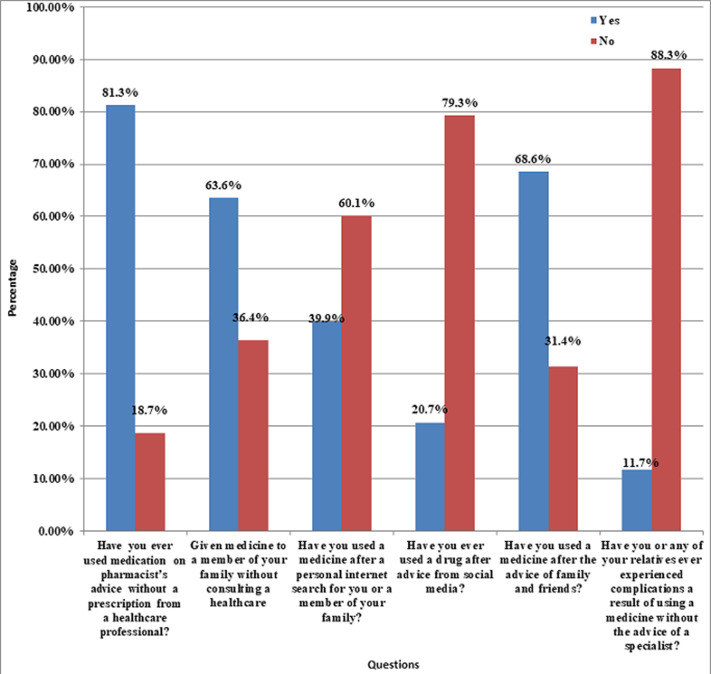
distribution of practices about medication without prescription from a healthcare professional

**Figure 2:**
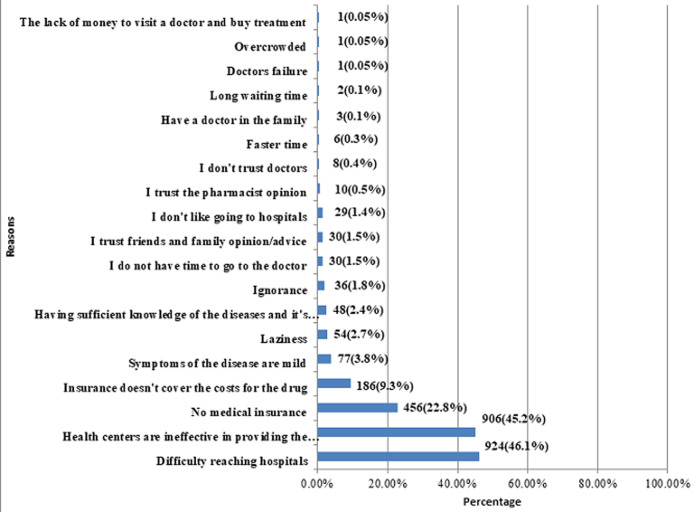
reasons for using medications without prescription

**Figure 3:**
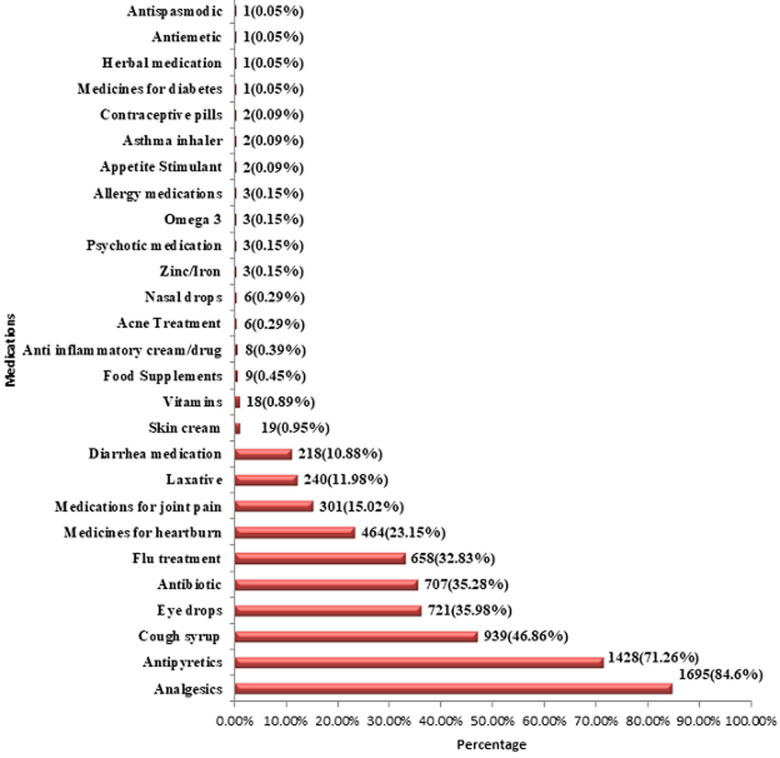
distribution of the type of medication used without prescription from a healthcare professional

Our study included a total of 2,004 participants who attended family clinics across the KSA. Most of the responders were Saudis (94%) and females (63%). Most of the patients belonged to the age bracket 20-29 (61.5%). Most of the respondents were University graduates (69.0%) and 50% of them belonged to the central KSA region. As for marital status, a substantial proportion was single (75.6%). Out of the married couples, 81.9% had no kids. A detailed description of the participants´ demographics is given in Table 1. Assessing the trends for self-medication, a substantial 1,630 (81.3%) participants responded to having used medication on pharmacist´s advice without a prescription from a healthcare professional. Furthermore, 63.6% of participants gave medications to their family members without consulting a qualified clinician. Advice from friends and family appeared to be the main stimulus for self-medication in 68.6% of cases, followed by information gathered from internet research (39.9%). A detailed analysis of self-medication practices is shown in Figure 1. Our research showed that 924 respondents (46.1%) found it difficult to reach a hospital. Another 45.2% considered the inefficiency of health centers in providing necessary care to be the main reason for self-medication. This is followed by a lack of medical insurance, insurance not covering the costs for the drugs, and symptoms of the disease being mild enough for not going to the hospital as the main triggers in 22.8%, 9.3% and 3.8% cases respectively. Figure 2 analyzes in detail the reasons for self-medication among the participants. Analgesics were the most commonly self-administered drugs (84.58%), followed by antipyretics (71.26%), cough syrups (46.86%), eye drops (35.98%), antibiotics (35.28%), flu medication (32.83%), heartburn medication (23.15%), medicines for joint pain (15.02%), and so on (Figure 3).

**Table 1:** descriptive statistics

Demographic characteristics of subjects		Frequency	Percentage
**Age groups(in years)**	Less than 20	445	22.2%
	20-29	1232	61.5%
	30-39	265	13.2%
	40-50	52	2.6%
	More than 50	10	.5%
**Gender**	Female	1262	63.0%
	Male	742	37.0%
**Educational level**	Primary	14	.7%
	Middle school	32	1.6%
	High school	468	23.4%
	Higher education	107	5.3%
	University level	1383	69.0%
**Nationality**	Non-Saudi	121	6.0%
	Saudi	1883	94.0%
**Region**	Central region	1005	50.1%
	Eastern region	256	12.8%
	Northern region	126	6.3%
	Southern region	173	8.6%
	Western region	444	22.2%
**Marital status**	Divorced	23	1.1%
	Married	461	23.0%
	Single	1516	75.6%
	Widow	4	.2%
**Kids**	1	137	6.8%
	2	74	3.7%
	More than2	152	7.6%
	No	1641	81.9%

Statistical significance was seen between gender (p = 0.001) and different reasons for self-administration with females more likely to self-administer compared to males. Statistical significance was also seen between age (p=0.001), marital status (p=0.001), and the number of kids (p=0.006). No statistical significance was achieved for education level and nationality (Table 2). As for assessing the correlation between different reasons for self-medication, statistical significance was achieved for lack of medical insurance (p=0.05), mild severity of symptoms (p=0.028), ignorance (p=0.000), not having time to go to the doctor (p=0.008), and not liking going to the hospital (p=0.000) only (Table 3). When different medications used were assessed against individual demographic variables, statistical significance was achieved between analgesics, laxatives, food supplements, and educational level with people with university-level education most likely to self-medicate (Table 4). Females were statistically more likely to use antipyretics, cough syrups, antibiotics, medications for joint pain, laxatives, diarrhea medication, and vitamins (Table 5). People between the 20-29 age bracket and singles were more likely to self-medicate as shown by Table 6and Table 7respectively.

**Table 2:** correlation between different factors and use of medication without prescription from a healthcare professional

Factors		Have you ever used a medication with your pharmacist's advice without a prescription from a healthcare professional?		P-Values
**No**	**Yes**
**Age**	20-29	229(18.6%)	1003(81.4%)	0.001 (Significant)
	30-39	30(11.3%)	235(88.7%)	
	40-50	7(13.5%)	45(86.5%)	
	Less than 20	104(23.4%)	341(76.6%)	
	More than 50	4(40%)	6(60%)	
**Gender**	Male	285(22.6%)	977(77.4%)	0.0001 (Significant)
	Female	89(12.0%)	653(88%)	
**Education level**	Primary	2(14.3%)	374(79.9%)	0.872 (Non-Significant)
	Middle school	5(15.6%)	27(84.4%)	
	High school	94(20.1%)	374(79.9%)	
	Higher education	21(19.6%)	86(80.4%)	
	University level	252(40%)	1131(60%)	
**Marital status**	Divorced	5(21.7%)	18(78.3%)	0.001 (Significant)
	Married	56(12.1%)	405(87.9%)	
	Single	312(20.6%)	1204(79.4%)	
	Widow	1(25%)	3(75%)	
**Kids**	No kids	330(20.1%)	1311(79.9%)	0.006 (Significant)
	1	16(11.7%)	121(88.3%)	
	2	10(13.5%)	64(86.5%)	
	More than 2	18(11.8%)	134(88.2%)	
**Nationality**	Non-Saudi	23(19%)	98(81%)	0.920 (Non-Significant)
	Saudi	351(18.6%)	1532(81.4%)	

Chi-square test was applied; P≤0.05(significant)

**Table 3:** correlation between different reasons and use of medication without prescription from a healthcare professional

Reasons	Have you ever used a medication with your pharmacist's advice without a prescription from a healthcare professional?		P-values
	No	Yes	
Difficulty reaching hospitals	169(18.3%)	755(81.7%)	0.692
Health centers are ineffective in providing the necessary care	170(18.6%)	736 (81.2%)	0.916
No medical insurance/insurance	71(15.6%)	385(84.4%)	0.05*
Insurance doesn't cover the costs for the drug	41(22%)	145(78%)	0.214
Symptoms of the disease are mild	7(9.1%)	70(90.9%)	0.028*
Laziness	10(18.5%)	44(81.5%)	0.978
Having sufficient knowledge of the diseases and it's treatment	5(10.4%)	43(89.6%)	0.138
Ignorance	16(44.4%)	20(55.6%)	0.000*
I do not have time to go to the doctor	0(0%)	30(100%)	0.008*
I trust friends and family opinion/advice	7(26.9%)	19(73.1%)	0.277
I don't like going to hospitals	14(48.3%)	15(51.7%)	0.000*
I trust the pharmacist opinion	0(0%)	10(100%)	0.128
I don't trust doctors	1(12.5%)	7(87.5%)	0.654
Faster time	0(0%)	6(100%)	0.24
Have a doctor in the family	0(0%)	3(100%)	0.406
Long waiting time	0(0%)	2(100%)	0.498
Doctors failure	0(0%)	1(100%)	0.632
Overcrowded	0(0%)	1(100%)	0.632
The lack of money to visit a doctor and buy treatment	0(0%)	1(100%)	0.632

Chi-square test was applied; P≤0.05(significant)

**Table 4:** correlation between education level and type of medications used without prescription from a healthcare professional

Type of Drugs	Educational Level					P-values
	UniversityLevel	HigherEducation	HighSchool	MiddleSchool	Primary	
Analgesics	1192(70.3%)	94(5.5%)	378(22.3%)	22(1.3%)	9(0.5%)	0.001*
Antipyretics	1004(70.3%)	70(4.9%)	324(22.7%)	22(1.5%)	8(0.6%)	0.250
Cough syrup	650(69.2%)	42(4.5%)	223(23.7%)	14(1.5%)	10(1.1%)	0.189
Eye drops	498(69.1%)	37(5.1%)	169(23.4%)	10(1.4%)	7(1.0%)	0.809
Antibiotics	493(69.7%)	39(5.5%)	159(22.5%)	8(1.1%)	8(1.1%)	0.297
Flu treatment	471(71.6%)	39(5.9%)	136(20.7%)	8(1.2%)	4(0.6%)	0.231
Medicines for heartburn	323(69.6%)	23(5%)	106(22.8%)	7(1.5%)	5(1.1%)	0.821
Medications for joint pain	214(71.1%)	13(4.3%)	64(21.3%)	5(1.7%)	5(1.7%)	0.179
Laxative	173(72.1%)	13(5.4%)	48(20%)	4(1.7%)	2(0.8%)	0.000*
Diarrhea medication	151(69.3%)	7(3.2%)	55(25.2%)	3(1.4%)	2(0.9%)	0.611
Skin cream	15(78.9%)	0(0%)	4(21.1%)	0(0%)	0(0%)	0.776
Vitamins	12(66.7%)	1(5.6%)	4(22.2%)	1(5.6%)	0(0.6%)	0.748
Food Supplements	5(55.6%)	1(11.1%)	1(11.1%)	2(22.2%)	0(0%)	0.000*
Anti-inflammatory cream/drug	7(87.5%)	0(0%)	1(12.5%)	0(0%)	0(0%)	0.841
Acne treatment	1(16.7%)	1(16.7%)	4(66.7%)	0(0%)	0(0%)	0.066
Nasal drops	3(50%)	0(0%)	3(50%)	0(0%)	0(0%)	0.631
Zinc/Iron	2(66.7%)	0(0%)	1(33.3%)	0(0%)	0(0%)	0.986
Psychotic medication	2(66.7%)	0(0%)	1(33.3%)	0(0%)	0(0%)	0.986
Omega 3	1(33.3%)	1(33.3%)	1(33.3%)	0(0%)	0(0%)	0.271
Allergy medication	2(66.7%)	0(0%)	1(33.3%)	0(0%)	0(0%)	0.986
Appetite stimulant	1(50%)	0(0%)	1(50%)	0(0%)	0(0%)	0.929
Asthma inhaler	1(50%)	0(0%)	1(50%)	0(0%)	0(0%)	0.929
Contraceptive pills	2(100%)	0(0%)	0(0%)	0(0%)	0(0%)	0.925
Medicine for diabetes	1(100%))	0(0%)	0(0%)	0(0%)	0(0%)	0.978
Herbal medication	0(0%)	0(0%)	1(100%)	0(0%)	0(0%)	0.512
Antiemetic	1(100%)	0(0%)	0(0%)	0(0%)	0(0%)	0.978
Antispasmodic	0(0%)	0(0%)	1(100%)	0(0%)	0(0%)	0.512

Chi-square test was applied; P≤0.05(significant)

**Table 5:** correlation between gender and type of medications used without prescription from a healthcare professional

Type of Drugs	Gender		P-values
	Female	Male	
Analgesics	1078(63.6%)	617(36.4%)	0.175
Antipyretics	874(61.2%)	554(38.8%)	0.010*
Cough syrup	537(57.2%)	402(42.8%)	0.000*
Eye drops	468(69.1%)	253(5.1%)	0.179
Antibiotics	376(53.2%)	331(46.8%)	0.000*
Flu treatment	428(65%)	230(35%)	0.179
Medicines for heartburn	241(51.9%)	223(48.1%)	0.000*
Medications for joint pain	165(54.8%)	136(45.2%)	0.001*
Laxative	124(51.7%)	116(48.3%)	0.000*
Diarrhea medication	92(42.2%)	126(57.8%)	0.000*
Skin cream	9(47.4%)	10(52.6%)	0.157
Vitamins	16(88.9%)	2(11.1%)	0.022*
Food Supplements	4(44.4%)	5(55.6%)	0.249
Anti-inflammatory cream/drug	5(62.5%)	3(37.5%)	0.978
Acne treatment	4(66.7%)	2(33.3%)	0.851
Nasal drops	4(66.7%)	2(33.3%)	0.851
Zinc/Iron	2(66.7%	1(33.3%)	0.895
Psychotic medication	2(66.7%	1(33.3%)	0.895
Omega 3	2(66.7%)	1(66.7%)	0.895
Allergy medication	3(100%)	0 (0%)	0.184
Appetite stimulant	2(100%)	0(0%)	0.278
Asthma inhaler	1(50%)	1(50%)	0.704
Contraceptive pills	2(100%)	0(0%)	0.278
Medicine for diabetes	1(100%))	0(0%)	0.443
Herbal medication	1(100%))	0(0%)	0.443
Antiemetic	0(0%)	1(100%)	0.192
Antispasmodic	0(0%)	1(100%)	0.192

Chi-square test was applied; P≤0.05(significant)

**Table 6:** correlation between age groups and type of medications used without prescription from a healthcare professional

Type of Drugs	Age groups					P-values
	<20 years	20-29years	30-39years	40-50years	>50 years	
Analgesics	376(22.2%)	1044(61.6%)	224(13.2%)	45(2.7%)	6(0.4%)	0.307
Antipyretics	311(21.8%)	875(61.3%)	193(13.5%)	41(2.9%)	8(0.6%)	0.628
Cough syrup	213(22.7%)	545(58%)	139(14.8%)	37(3.9%)	5(0.5%)	0.000*
Eye drops	168(23.3%)	438(60.7%)	95(13.2%)	19(2.6%)	1(0.1%)	0.456
Antibiotics	129(18.2%)	446(63.1%)	102(14.4%)	28(4%)	2(0.3%)	0.001*
Flu treatment	128(19.2%)	402(61.1%)	105(16%)	21(3.2%)	2(0.3%)	0.027*
Medicines for heartburn	77(16.6%)	268(57.8%)	97(20.9%)	20(4.3%)	2(0.4%)	0.000*
Medications for joint pain	64(21.3%)	179(59.5%)	37(12.3%)	17(5.6%)	4(1.3%)	0.001*
Laxative	40(16.7%)	145(60.4%)	39(16.2%)	16(6.7%)	0(0%)	0.000*
Diarrhea medication	44(20.2%)	123(56.4%)	35(16.1%)	15(6.9%)	1(0.5%)	0.000*
Skin cream	1(5.3%)	14(73.7%)	2(10.5%)	2(10.5%)	0(0%)	0.099
Vitamins	7(38.9%)	10(55.6%)	1(5.9%)	0(0%)	0(0%)	0.441
Food Supplements	0(0%)	7(77.8%)	2(22.2%)	0(0%)	0(0%)	0.520
Anti-inflammatory cream/drug	0(0%)	6(%)	2(0%)	0(0%)	0(0%)	0.539
Acne treatment	1(16.7%)	4(66.7%)	1(16.7%)	0(0%)	0(0%)	0.986
Nasal drops	1(16.7%)	4(66.7%)	1(16.7%)	0(0%)	0(0%)	0.989
Zinc/Iron	1(0%)	2(100%)	0(0%)	0(0%)	0(0%)	0.955
Psychotic medication	0(0%)	3(100%)	0(0%)	0(0%)	0(0%)	0.757
Omega 3	1(33.3%)	1(33.3%)	0(0%)	1(33.3%)	0(0%)	0.018*
Allergy medication	2(66.7%)	1(33.3%)	0(0%)	0(0%)	0(0%)	0.470
Appetite stimulant	0(0%)	1(50%)	1(50%)	0(0%)	0(0%)	0.627
Asthma inhaler	0(0%)	2(100%)	0(0%)	0(100%)	0(0%)	0.869
Contraceptive pills	0(0%)	2(100%)	0(0%)	0(0%)	0(0%)	0.869
Medicine for diabetes	0(0%)	1(100%)	0(0%)	0(0%)	0(0%)	0.960
Herbal medication	0(0%)	1(100%)	0(0%)	0(0%)	0(0%)	0.960
Antiemetic	0(0%)	1(100%)	0(0%)	0(0%)	0(0%)	0.960
Antispasmodic	0(0%)	1(100%)	0(0%)	0(0%)	0(0%)	0.960

Chi-square test was applied; P≤0.05(significant)

**Table 7:** correlation between marital status and type of medications used without prescription from a healthcare professional

Type of Drugs	Marital status				P-values
**Divorced**	**Married**	**Single**	**Widow**
Analgesics	22(1.3%)	379(22.4%)	1291(76.2)	3(0.2%)	0.186
Antipyretics	16(1.1%)	361(25.3%)	1049(73.5)	2(0.1%)	0.002*
Cough syrup	13(1.4%)	239(25.5%)	684(72.8%)	3(0.3%)	0.035*
Eye drops	6(0.8%)	155(21.5%)	559(77.5%)	1(1.1%)	0.420
Antibiotics	9(1.3%)	184(26%)	514(72.7%)	0(0%)	0.048*
Flu treatment	10(1.5%)	172(26.1%)	474(72%)	2(0.3%)	0.05*
Medicines for heartburn	4(0.9%)	153(33%)	305(65.7%)	2(0.4%)	0.000*
Medications for joint pain	5(1.7%)	75(24.9%)	219(72.8%)	2(0.7%)	0.133
Laxative	5(2.1%)	72(30.3%)	162(67.2%)	1(0.4%)	0.010*
Diarrhea medication	2(0.9%)	61(28%)	154(70.6%)	1(0.5%)	0.223
Skin cream	0(0%)	11(57.9%)	8(42.1%)	0(0%)	0.004*
Vitamins	1(5.6%)	3(16.7%)	14(77.8%)	0(0%)	0.329
Food Supplements	0(0%)	2(22.2%)	7(77.8%)	0(0%)	0.988
Anti-inflammatory cream/drug	0(0%)	3(37.5%)	5(62.5%)	0(0%)	0.795
Acne treatment	0(0%)	2(33.3%)	4(66.7%)	0(0%)	0.935
Nasal drops	0(0%)	1(25%)	3(75%)	0(0%)	0.996
Contraceptive pills	0(0%)	1(50%)	1(50%)	0(0%)	0.841
Zinc/Iron	2(66.7%)	0(0%)	1(33.3%)	0(0%)	0.000*
Psychotic medication	2(66.7%)	0(0%)	1(33.3%)	0(0%)	0.000*
Omega 3	0(0%)	1(33.3%)	2(66.7%)	0(0%)	0.976
Allergy medication	0(0%)	1(33.3%)	2(66.7%)	0(0%)	0.976
Appetite stimulant	1(50%)	0(0%)	1(50%)	0(0%)	0.000*
Asthma inhaler	0(0%)	2(100%)	0(0%)	0(0%)	0.082
Medicine for diabetes	0(0%)	0(0%)	1(100%)	0(0%)	0.956
Herbal medication	0(0%)	0(0%)	1(100%)	0(0%)	0.956
Antiemetic	0(0%)	0(0%)	1(100%)	0(0%)	0.956
Antispasmodic	0(0%)	0(0%)	1(100%)	0(0%)	0.956

## Discussion

Several themes can be delineated from the results of our study. First, the demographic details of self-medicators are not in keeping with what is reported by other studies which contend with the same topic. Indeed, two studies which were conducted in Ethiopia found that the majority of self-medicators belonged to the 25-44 and 30-45 age brackets respectively [5,13]. However, results from our study are in keeping with Saudi Arabian studies; one 2015 study found that most self-medicators were in the 23-33 age bracket [14]. These figures may reflect cultural differences between KSA and Ethiopia which account for the observation that self-medicators in KSA are generally younger. Another explanation for this observation is that the purchasing power amongst younger Saudi Arabians may be higher compared to their Ethiopian counterparts, as the former is more developed and has a more robust economy. As regards the reasons for self-medication, almost half of our participants faced challenges in access to healthcare and perceived health care delivery mechanisms as inefficient. This contrasts with the Ethiopian study mentioned previously, which found that the most commonly cited reason for self-medication was that the illness was perceived to be minor. In fact, only 5.1% of the participants in this study felt that healthcare institutions had no value to add, were too far or were inefficient [5]. In a society that more resembles the KSA closely, one study that was scoped towards Indians who lived in urban areas, found that participants most frequently self-medicated because they perceived that their illness was mild, or because they had time constraints which precluded seeking a consultation from a healthcare provider [15].

We observed a similar breakdown in the types of medications which were self-administered by our participants. Including our study, studies scoped to self-medicating behavior in various countries have found that analgesics are the most commonly self-administered drugs, followed by antipyretics and medications which provided symptomatic relief for upper respiratory tract symptoms (e.g. cough syrups) [12,16]. Several correlations were observed in our study. First, there was a statistically significant association between female gender and self-medication. Several studies have found that the prevalence of self-medication amongst women is moderate to high [17]. One study observed that the prevalence of self-medication amongst women may even increase during pregnancy [18]. Second, we also observed that participants with university-level education were more likely to self-medicate. The evidence regarding associations between self-medicating behavior and educational level is conflicted. In the study which looked at self-medication amongst pregnant women, the authors also observed that the odds-ratio of self-medication in women with secondary education was almost three-fold that of women with a high educational level [18]. In a similar Saudi Arabian study, the researchers observed that educational level was significantly associated with the mean knowledge score of medications [14]. It could be posited that participants with a higher degree of education, and therefore, knowledge of medications, could be more inclined to self-medicate as opposed to seeking professional advice from a physician. Similarly, statistically significant correlations were observed between self-medicating behavior and marital status as well as the number of children. This observation could be accounted for by the fact that young children frequently contract viral illnesses and upper respiratory tract infections which warrant symptomatic treatment with over-the-counter medications [19].

## Conclusion

This prospective cross-sectional study aimed to delineate current self-medication practices in KSA. The results suggest that the majority of Saudi Arabians engage in self-medicating behavior and rely on advice from friends and family most of the time, instead of consulting a health professional. Self-medication in KSA is associated with female gender, higher education level, being married and having children. These trends could be useful for Saudi health policymakers who seek to educate the public on self-medication in an attempt to mitigate adverse drug reactions and drug-drug interactions, especially amongst vulnerable populations such as children.

### What is known about this topic

- Self-medication is one of the major public health concerns worldwide, adopted by billions of people around the globe;
- Over-the-counter (OTC) drugs are the most frequently used medicines as self-medication;
- In Saudi Arabia, up to 81.4% of the general population has reported to use drugs without prescription at some point of their life.

### What this study adds

- Like other countries, analgesics, antipyretics and drugs for symptomatic relief for upper respiratory tract symptoms were among the most common self-medications in Saudi Arabia;
- In Saudi Arabia, top five causes of self-medication were difficulty to reach a hospital, inefficiency of health centers in providing necessary care, lack of medical insurance, insurance not covering the costs for the drugs, and symptoms of the disease being mild enough for not going to the hospital;
- The study findings are useful for Saudi health policy makers who seek to educate the public on self-medication in an attempt to mitigate adverse drug reactions and drug-drug interactions, especially among vulnerable populations such as children.
